# Differential peripheral immune dynamics underlie therapeutic response to chemotherapy and chemoimmunotherapy in triple-negative breast cancer

**DOI:** 10.1093/immhor/vlag049

**Published:** 2026-09-22

**Authors:** Zahra Mesrizadeh, Kavitha Mukund, Shankar Subramaniam

**Affiliations:** Department of Bioengineering, University of California San Diego, La Jolla, CA, United States; Department of Bioengineering, University of California San Diego, La Jolla, CA, United States; Department of Bioengineering, University of California San Diego, La Jolla, CA, United States; Departments of Cellular & Molecular Medicine, Computer Science & Engineering, and Data Science, University of California San Diego, La Jolla, CA, United States

**Keywords:** chemotherapy, immune checkpoint blockade, triple-negative breast cancer, tumor microenvironment

## Abstract

Triple-negative breast cancer (TNBC) is an aggressive breast cancer subtype with limited treatment options and response to immune checkpoint inhibitors. Tumor-infiltrating lymphocytes have been extensively studied; however, the integration of peripheral immune dynamics with mechanistic regulation underlying therapeutic response remain poorly defined. Here, we integrate immune-state modeling with pathway-level mechanistic inference to analyze single-cell RNA sequencing of PBMCs from patients with advanced TNBC treated with paclitaxel alone or in combination with the anti–PD-L1 Ab atezolizumab. This framework leverages treatment arm, longitudinal sampling, and clinical response to resolve coordinated immune programs across lymphoid and myeloid compartments. We identified distinct treatment- and response-specific states before and after treatment. Chemotherapy responders displayed pretreatment adaptive immune priming, whereas combination-therapy responders exhibited preexisting effector T-cell activity coupled with tumor PD-L1 expression. In contrast, chemotherapy nonresponders developed persistent post-treatment immune dysregulation in regulatory and terminal effector programs, whereas combination-therapy nonresponders demonstrated maladaptive remodeling of adaptive and innate compartments, including dysfunctional NK and metabolically reprogrammed myeloid populations. Across regimens, pathways involving protein translation, metabolic adaptation, and stress signaling emerged as shared modulators of response. These findings suggest that coordinated adaptive-innate immune dynamics underlie therapeutic efficacy, whereas systemic immune exhaustion and myeloid immunoregulation lead to resistance. Projection of these peripheral immune programs onto independent I-SPY2 showed concordant associations with tumor immune phenotypes and pathological complete response, supporting generalizability of the findings. Our study demonstrates the utility of an integrative approach for linking peripheral immune state organization with mechanistic insights, informing response in TNBC.

## Introduction

Triple-negative breast cancer (TNBC) is the most aggressive subtype of breast cancer, characterized by early relapse, poor prognosis, and an absence of targeted therapy. Despite the use of standard chemotherapy, approximately one-third of patients with stage II/III TNBC develop distant metastases within 2 to 3 years of diagnosis.^1^^,^^2^ The addition of immune checkpoint inhibitors (ICIs) to chemotherapy has improved survival in several malignancies with high tumor mutational burdens.^3^ This prompted investigation into their efficacy in TNBC, a relatively immunogenic subtype of breast cancer, enriched for tumor-infiltrating lymphocytes (TILs).

In the IMpassion130 trial, combination of atezolizumab (ATZ) with nanoparticle albumin-bound–paclitaxel (nab-PTX) improved progression-free survival in patients with PD-L1⁺ advanced TNBC (median progression-free survival: 7.5 vs. 5.0 months), leading to accelerated regulatory approval.^4^^,^^5^ However, follow-up studies, including IMpassion131, which paired ATZ with paclitaxel, and a separate international phase 3 trial (ALEXANDRA/IMpassion030), resulted in responders and nonresponders, even among patients with PD-L1⁺ disease.^2^^,^^6^ These mixed outcomes underscore a critical challenge in that only a subset of patients with TNBC achieve a durable response, highlighting the need to better define the system-level immune mechanisms that differentiate therapeutic response and resistance.

Although most efforts to date have focused on local immune responses within the tumor microenvironment (TME), effective antitumor immunity requires continuous interplay between the TME and the peripheral immune system. Circulating immune cells reflect systemic immune competence and, importantly, offer accessible and dynamic biomarkers of treatment response and disease progression. To date, peripheral immune profiling in breast cancer has largely relied on broad metrics such as neutrophil-to-lymphocyte ratio or lymphocyte-to-monocyte ratio,^7^^,^^8^ or limited immunophenotyping of major immune subsets using next-generation sequencing technologies such as single-cell RNA sequencing (scRNA-seq).^1^ Emerging studies in TNBC suggest systemic immune dysregulation is a defining feature. Patients with early-stage disease had fewer circulating B cells and elevated regulatory T-cell numbers compared with healthy control individuals,^9^ whereas patients with advanced disease demonstrated widespread immunosuppression marked by diminished CD4⁺ T cells, expansion of monocytes, and dysfunctional effector populations.^10^ These studies have largely lacked integrative, systems-level analyses capable of resolving the mechanistic basis of systemic immune response and resistance, particularly after chemotherapy versus combination therapy. Large neoadjuvant trials such as Investigation of Serial Studies to Predict Your Therapeutic Response With Imaging And Molecular Analysis 2 (I-SPY2) have generated clinically annotated transcriptomic datasets linking systemic immune signaling to treatment response, but these analyses largely rely on bulk immune signatures and do not resolve high-resolution immune subset states or coordinated peripheral immune programs.^11^^,^^12^

Here, we apply an integrative systems-level immune state analysis to scRNA-seq data from PBMCs (blood) and breast tissue with advanced TNBC treated with paclitaxel alone or in combination with anti–PD-L1 Ab ATZ (combination therapy).^13^ Our analysis highlights the transcriptional programs and immune-cell states associated with therapeutic response (in Rs and NRs) and treatment regimens (chemo- and combination therapy). Within peripheral immune cells (blood), chemotherapy Rs exhibited pretreatment adaptive immune priming, whereas combination therapy Rs showed preexisting effector adaptive immunity with innate lymphoid activation. Chemotherapy NRs developed persistent post-treatment immune dysregulation in regulatory and terminal effector programs, whereas combination therapy NRs showed post-treatment remodeling in adaptive and innate lymphoid compartments. Together, our study delineates coordinated systemic immune programs associated with therapeutic efficacy and resistance, providing insight into peripheral regulation in TNBC.

## Materials and methods

### Data overview

Published scRNA-seq data from Zhang et al.^7^ (Gene Expression Omnibus [GEO] accession GSE169246), which included 489,490 cells from 22 patients with advanced TNBC, spanning 75 tissue (primary/metastatic) and blood samples, were downloaded. The samples were collected pretreatment, 4 weeks after treatment, and at progression (Table S1). Patient P002 was excluded as indicated in the report by Zhang et al.^7^ The final dataset used for all downstream analyses included tissue samples (*n* = 15) and blood samples (*n* = 20). Patients had received either paclitaxel alone (chemotherapy; tissue, *n* = 7; blood, *n* = 10) or paclitaxel plus ATZ (combination therapy; tissue, *n* = 8; blood, *n* = 10), with documented treatment response available for most cases. Clinical response, including R and NR, TILs percentage, and PD-L1 scores, were obtained from the original study.

### Data filtering, scaling, and dimensionality reduction

Data were filtered by excluding cells according to the following criteria: more than 10% mitochondria read, fewer than 500 expressed genes, fewer than 500 detected transcripts, and a complexity (log10 genes/unique molecular identifier) of less than 80%. Following this quality control (QC), 432,837 cells remained for downstream analysis. Tissue-derived and PBMC samples were processed independently using Seurat (version 5.3.0), resulting in 2 data objects comprising 143,085 cells (tissue) and 289,752 cells (PBMCs) across 27,085 features or genes. Data scaling and dimensionality reduction were performed for each vignette, with the optimal dimensions for clustering identified using *ElbowPlot* (tissue, 30 principal components; PBMC, 31 principal components). The cells were clustered using the Louvain algorithm and visualized using Uniform Manifold Approximation and Projection (UMAP).

### Detecting cell types (cluster annotation)

Cell-type identities for each cluster detected were established using a 3-fold strategy: (i) SingleR with multiple reference datasets (DatabaseImmuneCellExpressionData, MonacoImmuneData, NovershternHematopoieticData); (ii) differential expression testing via Seurat’s *FindAllMarkers* (Wilcoxon rank-sum test, log fold change >0.25, minimum percentage of cells expressing the feature (min.pct) >0.25, Bonferroni correction *P* <0.05); and (iii) canonical marker expression and legacy knowledge. Markers were visualized using feature and bubble plots (top differentially expressed genes [DEGs]), enabling lineage assignment and transcriptional state assessment. Clustering yielded distinct immune populations in tissue samples, including 15 T-cell clusters, 5 B-cell clusters, 2 NK-cell clusters, and 8 myeloid clusters. PBMC samples revealed 13 T-cell clusters, 4 B-cell clusters, 5 NK-cell clusters, and 9 myeloid clusters.

### Profiling T-cell subsets

From the dataset, CD4⁺ and CD8⁺ T cells were extracted from both tissue and PBMC compartments for higher-resolution analysis. In tissue, a total of 90,286 CD4⁺/CD8⁺ T cells (composing 3 initial CD4⁺ clusters and 12 initial CD8⁺ clusters) were subsampled and re-clustered into 7 CD4⁺ and 14 CD8⁺ transcriptionally and functionally distinct subsets. From PBMCs, 120,213 CD4⁺/CD8⁺ T cells (from 9 initial CD4⁺ clusters and 14 initial CD8⁺ clusters) were subsampled and reclustered into 6 CD4⁺ and 15 CD8⁺ distinct subsets. Subsampling was performed to ensure balanced representation of patient samples and avoid bias from uneven cell recovery. Cell-type annotation was guided by established lineage and functional markers, including canonical naïve, central memory, effector, cytotoxic, proliferative, and regulatory T-cell signatures (Table S2).^1^^,^^2^^,^^14^^,^^15^ To assess the transcriptional characteristics of various T-cell subsets, we evaluated curated functional scores (eg exhaustion score, cytotoxicity score, anergy score), which were established based on a predefined set of genes obtained from a published pan-cancer T-cell study,^14^ using gene module scoring (the *AddModuleScore* function) in Seurat. Each score was calculated as the average expression level of the respective gene set.

### Profiling B-cell subsets

From 33,880 B cells (*n* = 4 original clusters), we created a subset from the original data object. They were reclustered and grouped into 10 functionally distinct B-cell states (plus 2 undefined), based on markers and pathway enrichment related to BCR signaling, MHC-II expression, IFN response, AP-1 transcriptional regulation, and metabolic/stress programs (Table S2).^1^

### Profiling NK-cell subsets

In tissue, a subset of 9,332 NK cells (*n* = 2 initial clusters) from the original dataset was reclustered into 8 transcriptionally and functionally distinct subsets based on DEG analysis and established classifications.^1^^,^^2^^,^^14^ In PBMCs, a subset of 55,745 NK cells (*n* = 5 initial clusters) was reclustered into 13 functional subsets using the same approach. Annotation incorporated canonical cytotoxic markers, activation and exhaustion profiles, and inflammatory gene signatures (Table S2).

### Profiling myeloid subsets

In tissue, a subset of 17,866 myeloid cells (*n* = 8 initial clusters) was reclustered into 23 transcriptionally distinct subsets. In PBMCs, a subset of 52,949 myeloid cells (*n* = 9 initial clusters) was reclustered into 9 subsets. Annotation identified classical (CD14⁺) and nonclassical (CD16⁺) monocyte populations, with further subdivision guided by transcriptional heterogeneity and functional gene programs, including inflammatory signaling, stress responses, and metabolic pathways (Table S2).

### Trajectory inference of CD8^+^ T cells

To reconstruct differentiation trajectories of CD8⁺ T cells, we used Monocle 3 (version 1.3.7).^16–20^ After dimensionality reduction with UMAP and clustering, we constructed cell trajectories by learning principal graphs using the *learn_graph* function with use_partition = FALSE to preserve global connectivity across all subsets. Default parameters were used because these yielded biologically coherent trajectories, with naïve T cells at the origin and expected branching toward effector and exhausted states. Pseudotime was assigned using *order_cells*, anchoring the root node based on naïve marker expression.

### Functional enrichment and protein-protein interaction/transcription factor network construction

Functional enrichment was identified via functional annotation clustering available through *clusterProfiler*^21^ or *Enrichr*^22^ in R. Gene Ontology (biological process and molecular function) pathway analyses were performed using *clusterProfiler’s EnrichGO* function. A network of high confidence (>0.70) human protein-protein interaction (PPI) was downloaded from STRING database, version 12,^23^ containing 15,588 proteins and 236,930 interactions. Additionally, transcription factor (TF)-target data downloaded from the TRRUST-db^24^ was mapped onto the PPI interaction network to generate a custom PPI/TF network.

Therapy-, response, and time point–specific PPI/TF sub-networks were generated using DEGs from adaptive and innate immune subsets described earlier in the “Cell-type annotation”. In blood these included the following:

- adaptive chemotherapy responder (pretreatment): 746 proteins, 8,108 interactions
- innate chemotherapy responder (pretreatment): 986 proteins, 6,876 interactions
- adaptive chemotherapy nonresponder (post-treatment): 176 proteins, 4,078 interactions
- innate chemotherapy nonresponder (post-treatment): 232 proteins, 3,560 interactions
- adaptive combination responder (pretreatment): 361 proteins, 3,560 interactions
- innate combination responder (post-treatment): 2,089 proteins, 13,919 interactions
- adaptive combination nonresponder (post-treatment): 103 proteins, 1,178 interactions
- innate combination nonresponder (post-treatment): 731 proteins, 5,101 interactions

In tissue, these included

- adaptive chemotherapy responder (pretreatment): 211 proteins, 1,139 interactions
- adaptive chemotherapy responder (post-treatment): 41 proteins, 274 interactions
- adaptive chemotherapy nonresponder (post-treatment): 239 proteins, 3,280 interactions
- adaptive combination responder (pretreatment): 166 proteins, 2,579 interactions
- adaptive combination responder (post-treatment): 38 proteins, 101 interactions
- adaptive combination nonresponder (pretreatment): 162 proteins, 2,066 interactions

Each network was refined by selecting DEGs, identifying associated TFs, and extracting their 1-step PPI neighbors. All networks were visualized, annotated, and analyzed using Cytoscape.^25^

### Statistical modeling of blood-tissue expression relationships

We used linear mixed-effects models to quantify the relationship between blood and tissue gene expression across immune subsets. Models were fitted in R using the lme4 package. For each gene, expression in tissue was modeled as a function of blood expression, immune cell subsets, and their interaction, with a random intercept for the patient. Models were fit separately for pre- and post-treatment data using restricted maximum likelihood. Model performance was summarized by marginal and conditional *R*^2^ values using the performance package. To assess robustness, we performed leave-1-patient-out analyses by iteratively removing 1 patient, refitting the model, and recording (i) changes in restricted maximum likelihood criterion values, (ii) shifts in fixed-effect estimates, and (iii) variance components. The fixed-effect regression coefficient (β) quantified the expected change in tissue gene expression per 1-unit increase in blood gene expression, adjusted for immune-cell subset composition.

### External validation using I-SPY2 peripheral transcriptomic data

ISPY2 clinical trial raw data were downloaded from GEO accession GSE194040, which includes pretreatment tumor gene expression profiles and clinical annotations for patients enrolled across multiple neoadjuvant treatment arms.^11^ Analyses were restricted to the paclitaxel arm to ensure treatment comparability with the discovery cohort. Gene expression data generated on Agilent microarray platforms (GPL20078) were downloaded from GEO. Probe-level measurements were mapped to gene symbols using platform annotations and collapsed to gene-level expression using median aggregation. Genes with zero variance across samples were excluded. Peripheral immune subset gene signatures derived from PBMC profiling in this study were projected onto the I-SPY2 cohort using single-sample gene set enrichment analysis implemented via the Gene Set Variation Analysis framework, generating per-sample immune program scores. Associations between immune subset scores and pathological complete response were evaluated using logistic regression. Multivariate relationships were assessed using Least Absolute Shrinkage and Selection Operator (LASSO)-regularized logistic regression with cross-validation, and model robustness was evaluated using permutation testing.

## Results

### Identification of key mechanisms driving peripheral blood immune-cell dynamics across treatment and response

**Figure 1:**
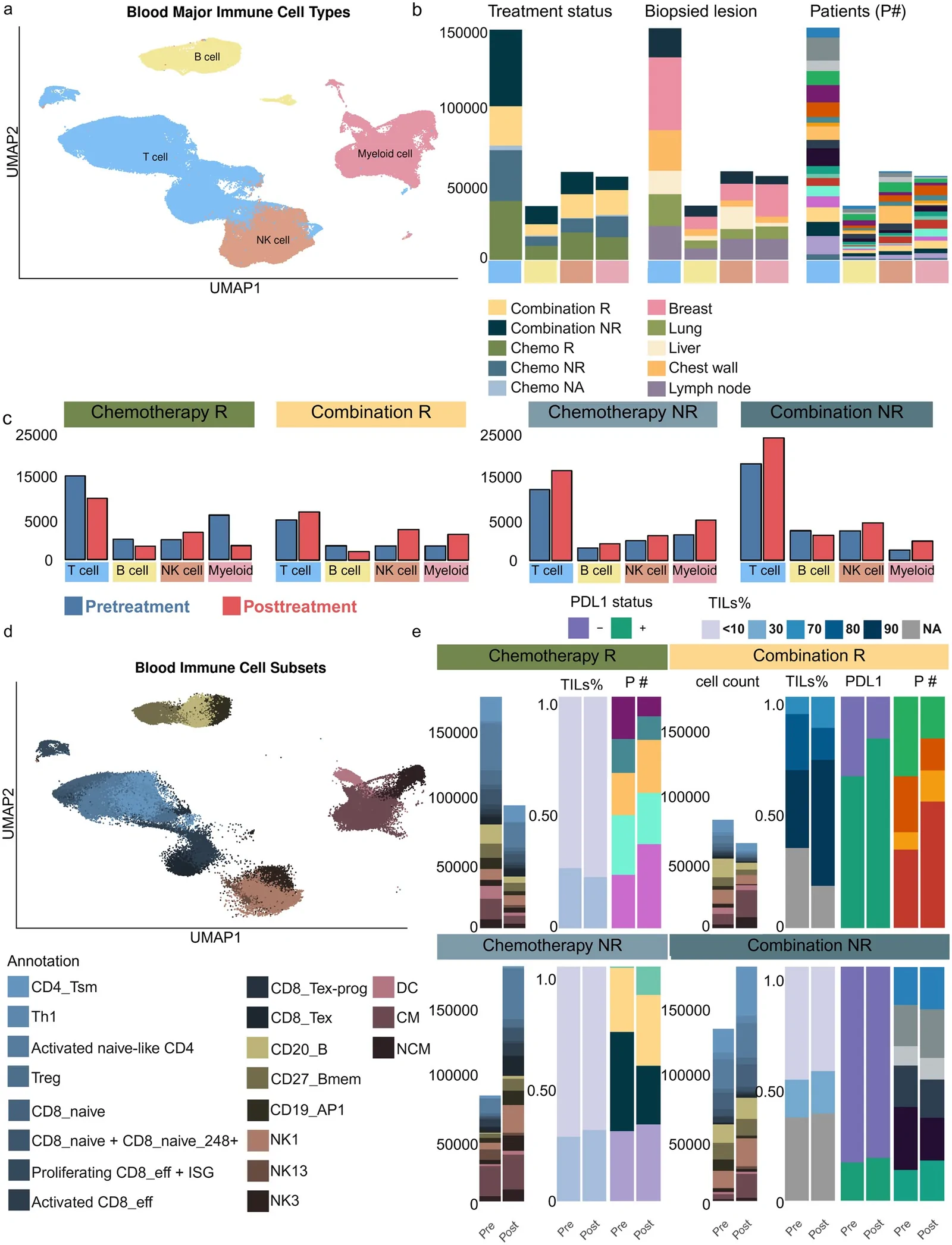
Comparative immune landscape of blood samples under different treatment conditions. (A) UMAP plots depict major immune cell types in blood, highlighting T cells (blue), B cells (yellow), monocytes (pink), and NK cells (brown). (B) Compositions of major immune cell types in treatment and response groups, biopsied lesions, and individual patients (P#). Colors in the patient panel denote individual patients. (C) Bar plots quantifying immune cell frequencies, illustrating treatment-associated shifts in immune cell distribution. (D) UMAP plot depicting blood immune subsets, with colors denoting functionally annotated subsets. (E) Integrated paired pretreatment (Pre) and posttreatment (Post) bar plots showing frequencies of functional immune subsets, proportions of TILs, PD-L1⁺ (PDL) cells, and patient group identifiers (P#), stratified by responder (R) and nonresponder (NR) status. This panel links peripheral immune subset dynamics with intratumoral immune contexture. Colors in the patient panel denote individual patients.

Overall, 143,085 and 289,752 high-quality cells across 4 major cell types were identified after rigorous quality control across tissue and blood, respectively. To systematically assess treatment-associated immune alterations, we analyzed changes in circulating immune-cell frequencies after treatment compared with before treatment across each treatment and response group. Given biased patient-specific clustering of immune-cell types in tissue samples, except for T cells, we focused most of our dynamic analysis on blood (Fig. 1A, B; Fig. S1A, B). In chemotherapy-treated patients, responders exhibited a post-treatment decrease in B cells, T cells, and myeloid cells, alongside an increase in NK cells (Fig. 1C). In contrast, nonresponders showed a global increase across all immune cell types after treatment. In the combination-therapy group, both responders and nonresponders had increased immune cell populations post-treatment, except B cells, which decreased in both groups (Fig. 1C).

To resolve immune programs associated with these frequency shifts, we next stratified major immune lineages into functional sub-clusters based on gene expression programs (eg activation, exhaustion, migration) using known immunological signatures (Fig. 1D).^1^^,^^2^ We focused on subtypes that were representative across patients and differentially enriched by treatment response. All comparisons were made between post- and pretreatment samples within each group. Although stromal TIL levels and peripheral immune-cell counts often only moderately correlate in patients with TNBC,^3^ declining TILs after chemotherapy have been linked to treatment response, whereas persistently high post-treatment TILs levels can lead to poor outcomes.^4^ Furthermore, the predictive value of TILs and PD-L1 expression in informing ICI efficacy is well-established.^5^

In this dataset, analysis of response-specific TIL levels demonstrated that chemotherapy-treated patients, irrespective of response, had consistently low TIL levels (Fig. 1E). Notably, tissue samples from chemotherapy responders included both primary breast and metastatic lesions (stage IV), whereas tissue samples from chemotherapy nonresponders were exclusively derived from primary breast tumors with stage II–III disease (Fig. S1D). When integrating peripheral immune profiling, functional subsets defined from PBMCs decreased only in responders after treatment in chemotherapy, consistent with systemic immune activation and potential redistribution toward tumor or other tissues (Fig. 1E). In combination therapy, responders, predominantly from lymph node lesions (stage IV), also had post-treatment decreases in circulating functional subsets, particularly in high TIL and PD-L1⁺ tumors, further suggesting immune localization (Fig. 1D, E; Fig. S1D). In contrast, combination nonresponders had post-treatment increases in peripheral immune subsets despite low TIL levels, indicating a disconnect between systemic activation and intratumoral recruitment, consistent with immune dysfunction or exclusion (Fig. 1E; Fig. S1D).

Using linear mixed-effects modeling, we demonstrated that peripheral blood gene expression robustly predicted tissue-level expression (pretreatment β = 0.49, *t* = 44.9; post-treatment β = 0.57, *t* = 54.6; *P* < 0.001), with significant interactions indicating that this relationship varies across immune functional subsets (Fig. S1E). These results support the hypothesis that systemic immune signatures reflect and are associated with tumor immune states in a subset-dependent manner. To further understand these dynamics, we examined changes within the lymphoid and myeloid subsets across treatment and response groups.

### Lymphoid compartments

The lymphoid compartment showed distinct composition and mechanistic patterns across treatments and responses. In chemotherapy responders, pretreatment priming of adaptive immunity was a key feature, whereas post-treatment persistence of effector or exhaustion states characterized resistance, suggesting these patients might benefit from immunotherapy. In combination therapy, pretreatment effector or exhaustion signatures were associated with response, and resistance was linked to dysregulated adaptive immunity.

#### CD4 T-cell subsets: pretreatment immune priming characterizes responders

**Figure 2:**
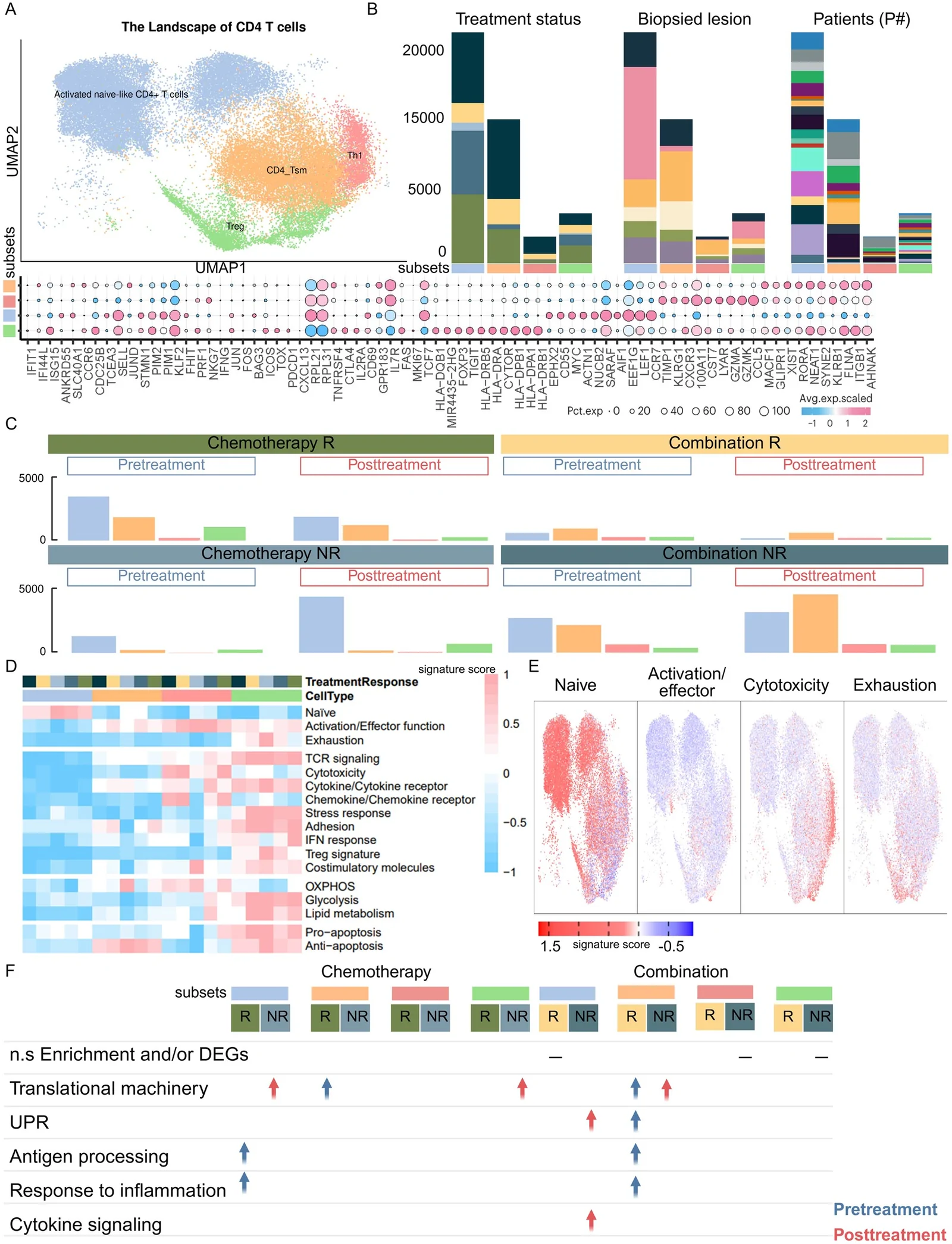
Comprehensive landscape of CD4⁺ T-cell subsets in blood samples and their distribution across conditions. (A) UMAP representation of CD4⁺ T-cell sub-clusters in blood, colored by distinct functional states (top). Bubble plot showing representative marker gene expression across clusters (bottom). Additional marker genes are listed in Table S2. (B) Stacked bar plots of CD4⁺ T-cell sub-clusters across treatment and response groups, biopsied lesions, and individual patients, using color annotations consistent with Fig. 1B. (C) Bar plots comparing the distribution of CD4⁺ T-cell sub-clusters before and after treatment across samples, colored by functional states. (D) Heat map of 16 curated gene signature scores across CD4⁺ T-cell clusters.^23^ (E) Expression of 4 representative gene signatures selected from (D). (F) Table showing enriched pathways or functional gene signatures associated with CD4⁺ T-cell sub-clusters, stratified by condition (blue: enriched in pretreatment; red: enriched in post-treatment). This figure highlights the transcriptional heterogeneity of CD4⁺ T cells in blood, their patient-specific variability, and dynamic changes in sub-cluster composition and function in response to treatment. Pathway enrichment analyses point to key functional programs shaping peripheral CD4⁺ T-cell dynamics. n.s., not significant; NR, nonresponder; R, responder; UPR, unfolded protein response.

CD4^+^ T-cell subsets coordinate adaptive immune responses through helper, regulatory, and memory programs that can either support antitumor immunity or promote immune suppression within the TME. Of the 6 CD4^+^ T-cell clusters identified (Table S2), 4 functional clusters (activated naïve-like CD4, regulatory CD4 [Treg], Th1, and T stem-like memory [CD4_Tsm]) that were representative across patients were selected for detailed analysis (Fig. 2A, B). In the chemotherapy group, responders exhibited a pretreatment increase in activated naïve-like CD4 with increased chemokine receptor, IFN response, adhesion, and metabolic scores (Fig. 2C–E; Fig. S2A, B). Differential analysis further showed enrichment of IFN-driven inflammatory programs and Ag processing, consistent with enhanced immune priming (Fig. 2F; Fig. S2C).^6^ Notably, this aligns with prior observations by Zhang et al.,^7^^,^^8^ who identified a naïve CD4 cluster (CD4 Tn_CCR7) as predictive of response to both PTX and nab-PTX. CD4_Tsm subsets also had increased stress response and glycolysis scores, suggesting metabolically active memory-like cells responding to sustained immune stimulation (Fig. 2C–F; Fig. S2A). Treg cell numbers also were increased before treatment, with stress response, metabolic programs, and pro-apoptotic scores consistent with regulatory activation before treatment (Fig. 2C, F; Fig. S2A, C). In the combination-therapy group, responders also had a pretreatment increase in activated naïve-like CD4 with elevated lipid metabolism scores, alongside CD4_Tsm subsets enriched for stress response, oxidative phosphorylation (OXPHOS), glycolysis, and pro-apoptotic scores, mirroring the metabolically active immune priming signatures observed in the chemotherapy groups (Fig. 2C; Fig. S2A, B).

#### CD4 T-cell subsets: metabolic dysregulation characterizes nonresponders

In contrast with responders, nonresponders lacked evidence of pretreatment immune priming. In chemotherapy nonresponders, treatment induced expansion of activated naïve-like CD4 with increased IFN response, OXPHOS, glycolysis, and pro-apoptotic scores, alongside Tregs cells enriched for OXPHOS score, indicating a shift toward regulatory and pro-tumor activation (Fig. 2C–E; Fig. S2A, B). Differential analysis further showed enrichment of translational machinery in both CD4 subsets, supporting a regulatory and metabolically active but potentially ineffective immune state (Fig. 2F; Fig. S2C). Notably, although our observations are from peripheral blood, they align with findings in the Zhang et al. study.^7^^,^^8^ Zhang et al. reported increased Treg cell frequencies after chemotherapy in tumor-tissue nonresponders, suggesting a systemic and intratumoral response associated with treatment resistance.^7^^,^^8^

In combination-therapy nonresponders, post-treatment CD4_Tsm expanded with high cytotoxicity, IFN response, glycolysis, and pro-apoptotic scores alongside enrichment for translational programs. Activated naïve-like CD4 exhibited high cytotoxicity, cytokine receptor, adhesion, and metabolic scores (Fig. 2C–E; Fig. S2A, B), whereas differential analysis demonstrated enrichment of cytokine signaling and unfolded protein response pathways, indicative of cellular stress response rather than effective immune priming (Fig. 2F; Fig. S2). Increased frequencies of Treg cells with elevated IFN response scores, together with Th1 cells enriched for IFN response and anti-apoptotic scores, reflected an imbalanced and potentially dysfunctional CD4 T-cell response (Fig. 2C–E; Fig. S2A, B).

#### CD8 T-cell subsets: pretreatment naïve and effector expansion in chemotherapy and terminal differentiation in combination therapy characterize responders

**Figure 3:**
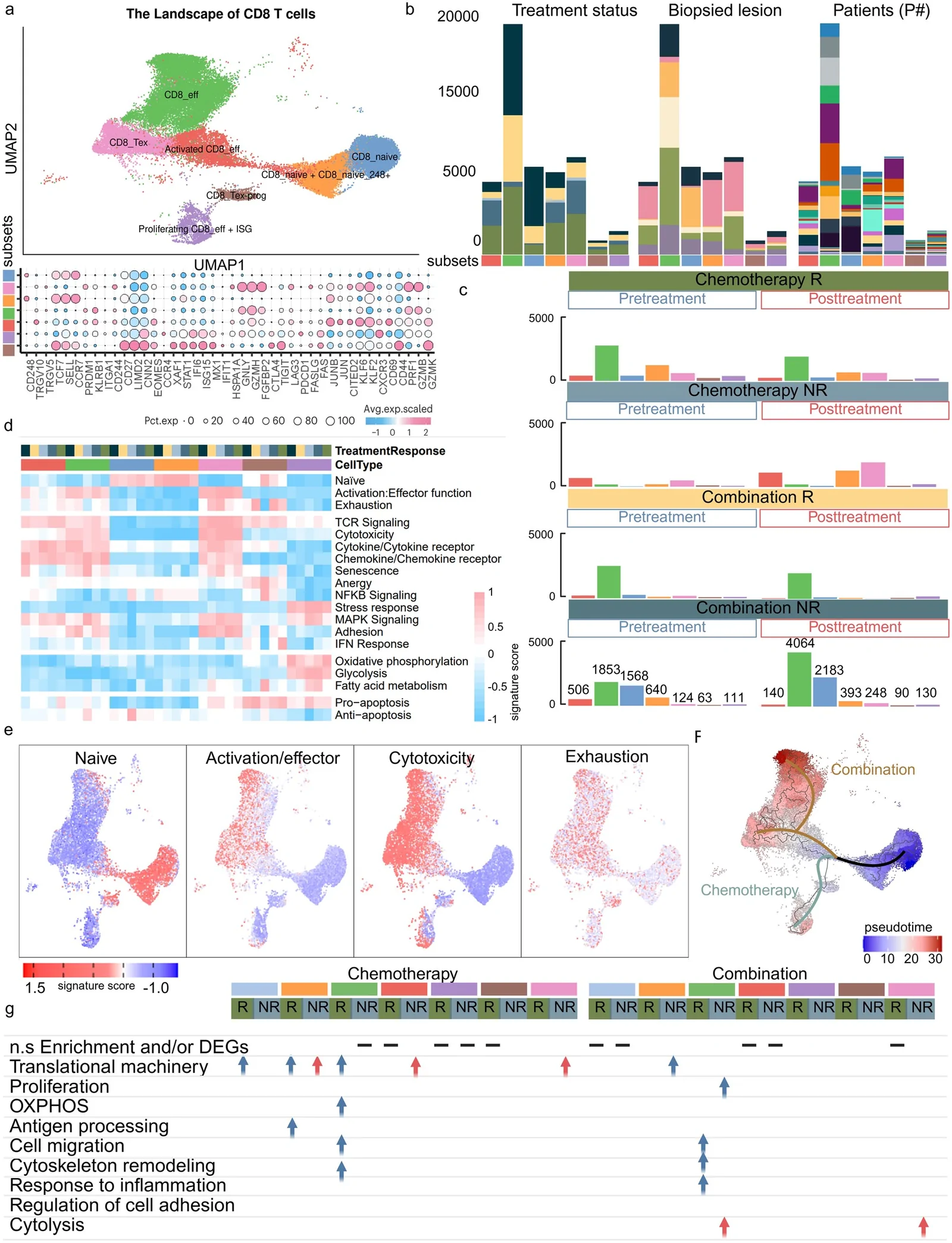
Comprehensive landscape of CD8⁺ T-cell subsets in blood samples and their distribution across conditions. (A) UMAP representation of CD8⁺ T-cell sub-clusters in blood, colored by distinct functional states (top). Bubble plot showing representative marker gene expression across clusters (bottom). Additional marker genes are listed in Table S2. (B) Stacked bar plots of CD8⁺ T-cell sub-clusters across treatment and response groups, biopsied lesions, and individual patients (P#), using color annotations consistent with Fig. 1B. (C) Bar plots comparing the composition of CD8⁺ T-cell sub-clusters in blood before and after treatment across multiple samples, colored by distinct functional states. (D) Heat map illustrating the expression of 19 curated gene signatures across CD8⁺ T-cell clusters. (E) Expression of 4 representative gene signatures selected from (D). (F) Monocle 3 trajectory analysis of CD8⁺ T-cell differentiation revealing 3 main divergent trajectories. Cells are color-coded by pseudo time. (G) Table representation of enriched pathways or functional signatures associated with CD8⁺ T-cell sub-clusters, stratified by condition. (Blue: enriched in pretreatment; red: enriched in post-treatment). This figure illustrates the heterogeneity of CD8⁺ T cells in peripheral blood, their inter-patient variability, and dynamic reprogramming in response to treatment. Enrichment analysis highlights context-specific pathways associated with CD8⁺ T-cell functional transitions. avg.exp., average expression; n.s., not significant; NR, nonresponder; pct.exp., percentage of cells expressing the gene; R, responder.

CD8^+^ T cells mediate cytotoxic antitumor immunity, and chronic stimulation can drive exhaustion and impaired effector function. Of the 15 CD8^+^ T-cell clusters identified (Table S2), 7 functional clusters (CD8_naïve, CD8_naïve + CD8_naïve_248^+^, effector cells [CD8_eff], activated CD8_eff, exhausted T cells [CD8_Tex], proliferating CD8_eff + IFN-stimulating genes [ISG], and CD8_Tex-prog) that had cell contributions from all patients were selected for detailed analysis (Fig. 3A, B). In the chemotherapy group, responders exhibited a pretreatment increase in several naïve clusters, including CD8_naïve enriched for cytotoxicity and chemokine receptor scores, and CD8_naïve + CD8_naïve_248^+^ enriched for cytotoxicity, IFN response, OXPHOS, and glycolysis scores, alongside differential enrichment in translational and Ag processing programs (Fig. 3C–G; Table S2, Fig. S3A–C). The frequencies of effector CD8^+^ subsets, including CD8_eff, activated CD8_eff, proliferating CD8_eff + ISG, and CD8_Tex-prog, were elevated, highlighting the association of transitional and cytotoxic states with chemotherapy response (Fig. 3C). CD8_eff cells had increased TCR signaling, glycolysis, and pro-apoptotic scores (Fig. 3D, E; and Fig. S3A, B). Differential analysis further confirmed upregulation of translational machinery, cell migration, cytoskeleton remodeling, and OXPHOS (Fig. 3G; Fig. S3C). Activated CD8_eff were enriched for naïve, adhesion, IFN response, OXPHOS, and glycolysis scores (Fig. 3D, E; Fig. S3A). Proliferating CD8_eff + ISG subsets were marked by high stress, OXPHOS, and metabolic scores, and CD8_Tex-prog had increased adhesion scores (Fig. 3D, E; Fig. S3A).

Expansion of effector and transitional CD8^+^ T cells with heightened TCR signaling and metabolic activity is consistent with Ag-experienced cytotoxic immune programs during chemotherapy, a pattern consistent with established models with metabolic reprogramming in activated CD8^+^ T cells.^9^ In combination-therapy responders, CD8^+^ T-cell dynamics had distinct features compared with chemotherapy alone. Responders had increased frequencies of the CD8_naïve subset, alongside more effector subsets (CD8_eff, activated CD8_eff, and CD8_Tex), indicating a functionally engaged and terminally differentiated cytotoxic program (Fig. 3C–E; Fig. S3B), although CD8_eff cells showed increased activation or effector, exhaustion, TCR signaling, cytotoxicity, cytokine receptor, metabolic, and pro-apoptotic scores (Fig. 3D, E; Fig. S3A).

Differential analysis revealed similar enrichment in cell migration and cytoskeletal remodeling pathways, with combination therapy uniquely adding inflammatory response programs (Fig. 3G; Fig. S3C). Notably, CD8_Tex cells in the combination setting had elevated OXPHOS scores (Fig. 3D, E; Fig. S3A). These systemic profiles are consistent with previously reported intratumoral CD8⁺CXCL13⁺ exhausted T cells,^7^ suggesting a coordinated peripheral and tissue immune engagement.

#### CD8 T-cell subsets: terminal differentiation and dysregulated cytotoxic programs in nonresponders

In chemotherapy nonresponders, treatment induced expansion of CD8_naïve + CD8_naïve_248^+^, characterized by high naïve scores (Fig. 3C–E; Fig. S3A) and enrichment in translational pathways (Fig. 3G; Fig. S3). Additionally, effector and terminal subsets including CD8_eff, activated CD8_eff, proliferating CD8_eff + ISG, and CD8_Tex were expanded, reflecting a skew toward cytotoxic and fully differentiated exhausted phenotypes (Fig. 3C; Fig. S3B). These subsets showed strong enrichment for cytotoxicity, chemokine receptor, OXPHOS, and glycolysis scores, whereas proliferating CD8_eff + ISG increased IFN response and OXPHOS scores (Fig. 3D, E; Fig. S3A). Differential analysis showed enrichment of translational machinery associated with Activated CD8_eff and CD8_Tex (Fig. 3G; Fig. S3C). In the combination-therapy group, nonresponders exhibited a distinct pattern of immune remodeling compared with chemotherapy. In pretreatment, CD8_naïve + CD8_naïve_248^+^ subsets were elevated and had high anergy scores (Fig. 3C–E; Fig. S3A, B) and enrichment in translational pathways (Fig. 3G; Fig. S3C). Additionally, activated CD8_eff subsets showed high activation, chemokine receptors, and IFN response scores, suggesting dampened effector function prior to treatment (Fig. 3 C–E; Fig. S3A). Post-treatment increases were observed in CD8_naïve cells with high naïve scores, CD8_eff subsets enriched for activation, cytotoxicity, cytokine receptors, and adhesion scores, and CD8_Tex with cytotoxicity and adhesion scores (Fig. 3C–E; Fig. S3A, B). Differential analysis confirmed strong enrichment of cytolysis-associated genes in effector subsets (Fig. 3G; Fig. S3C). These results indicated ineffective or dysregulated T-cell activation in the combination nonresponder group.

Pseudo time analysis of CD8⁺ T cells revealed 2 main trajectories: (i) from naïve through activated effector states toward transitional populations (Tex-prog, CD8_eff + ISG), and (ii) toward fully differentiated Tex and cytotoxic effector subsets (Fig. 3F). In the chemotherapy group, cells were primarily enriched in the transitional branch, whereas combination-therapy cells predominantly occupied the terminally differentiated branch, reflecting more complete effector maturation under combination therapy. Notably, the apparent differences between responders and nonresponders within each treatment group were largely explained by temporal dynamics, with responders enriched in these states at pretreatment and nonresponders at post-treatment.

#### B cells: translationally active and primed at baseline in responders

**Figure 4:**
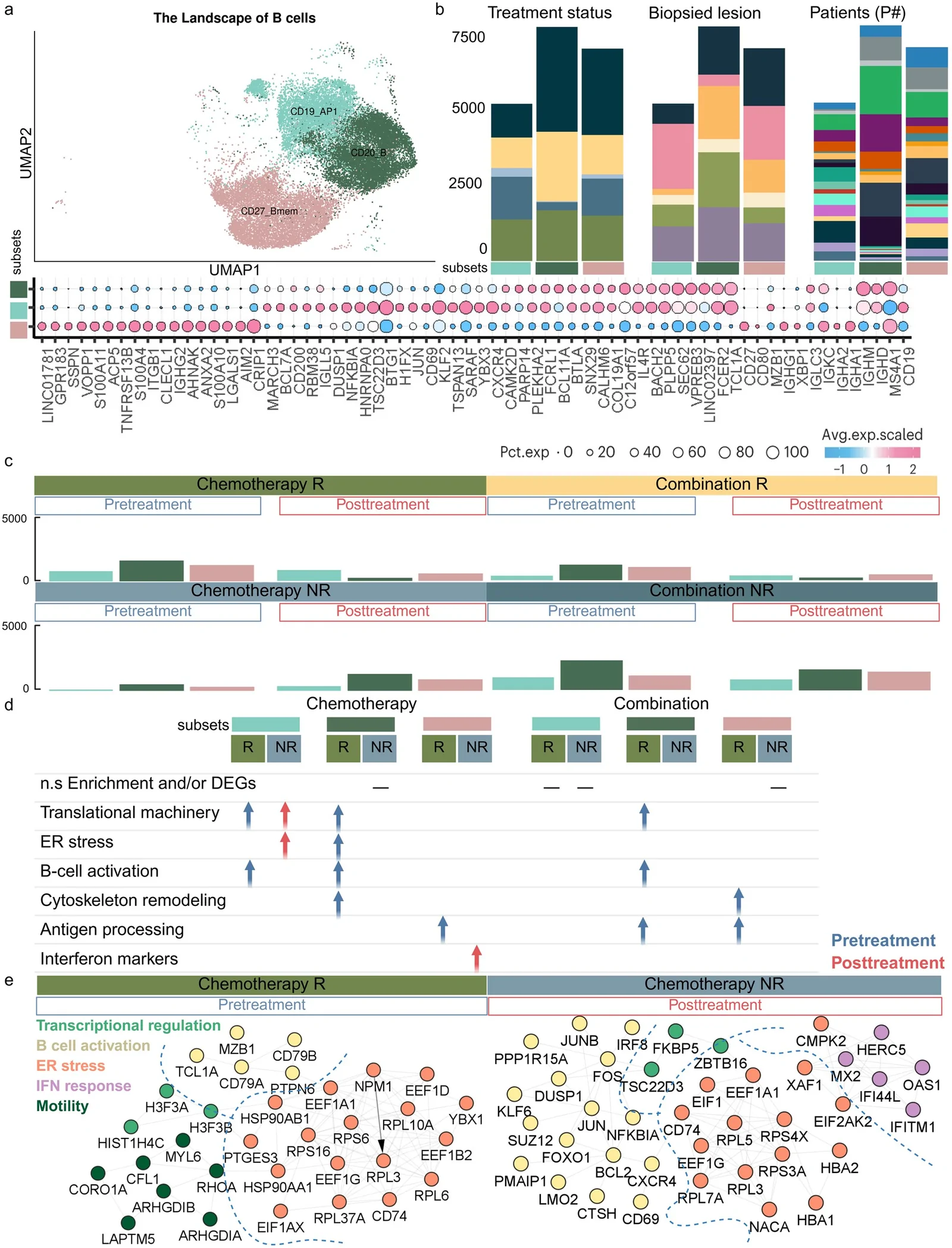
Comprehensive landscape of B-cell subsets in blood samples and their distribution across conditions. (A) UMAP representation of B-cell sub-clusters in blood, colored by distinct functional states. The bubble plot shows representative marker gene expression across clusters. Additional marker genes are listed in Table S2. (B) Stacked bar plots of B-cell sub-clusters across treatment and response groups, biopsied lesions, and individual patients (P#), using color annotations consistent with Fig. 1B. (C) Bar plots comparing the composition of B-cell sub-clusters in blood before and after treatment across multiple samples, colored by functional states. (D) Table summarizing enriched pathways or functional signatures associated with B-cell sub-clusters, stratified by condition. Blue: enriched in pretreatment; red: enriched in post-treatment. (E) PPI networks highlight distinct mechanisms enriched in the chemotherapy responder (R) pretreatment (right) and chemotherapy nonresponder (NR) post-treatment (left) groups. This figure illustrates the heterogeneity of B cells in peripheral blood, highlighting patient-specific variation and dynamic changes in sub-cluster composition and functional states in response to treatment. Enrichment and network analyses underscore key signaling pathways associated with B-cell activity across clinical conditions. avg.exp., average expression; ER, endoplasmic reticulum; n.s., not significant; NR, nonresponder; pct.exp., percentage of cells expressing the gene; R, responder.

B cells contribute to antitumor immunity through Ag presentation, Ab production, and modulation of T-cell responses, although regulatory B-cell states can also support immune suppression.^26^ Of the 12 B-cell clusters identified (Table S2), 3 functional clusters (CD19_B cells, CD20_B cells, and memory B cells [CD27_Bmem]) that were representative across patients were selected for detailed analysis (Fig. 4A, B). In the chemotherapy group, responders had a pretreatment increase in B-cell populations, including CD20^+^ B cells, CD19^+^_AP1, and CD27^+^ memory B cells, enriched for cytoplasmic translation (Fig. 4D; Fig. S3D), suggesting increased activation and protein synthesis.^10^ Pretreatment PPI network analysis of these populations revealed enrichment of ribosomal and translational regulators, stress proteins, and BCR signaling components (CD79A/B, PTPN6), indicating a transcriptionally and translationally engaged B-cell state in chemotherapy responders (Fig. 4E). Notably, although these results are derived from peripheral blood, they align with prior findings demonstrating that B cells within the TME were predictive of response to chemotherapy: decreasing in paclitaxel-treated responders but increasing in those treated with nab-paclitaxel plus ATZ.^8^ Together, these data suggest that B-cell activation signatures in circulation and tissue are associated with treatment responsiveness. In combination therapy responder, pretreatment B-cell populations displayed distinct dynamics compared with chemotherapy. CD20^+^ B cells were enriched for translational machinery, B-cell activation, and Ag processing, whereas CD27^+^ memory B cells were enriched for cytoskeleton remodeling and Ag processing, and CD19^+^_AP1 were elevated in pretreatment but lacked significant mechanism enrichment, suggesting differential B-cell dynamics compared with chemotherapy (Fig. 4D; Fig. S3D).

#### B cells: stress-regulated and dysregulated programs in nonresponders

In chemotherapy nonresponders, treatment induced expansion of B-cell populations, including CD20^+^ B cells, CD19^+^_AP1, and CD27^+^ memory B cells, which were enriched for translational machinery (Fig. 4C, D). PPI network analysis highlighted ribosomal and translational regulators, stress- and apoptosis-related proteins, and IFN-inducible genes. Immune checkpoint and regulatory-associated genes, including *CD69*, *CXCR4*, *JUN*, *IRF8*, and *ZBTB16*, were also notable, suggesting a multifaceted stress-regulatory B-cell response (Fig. 4E). In combination-therapy nonresponders, pretreatment frequencies of B-cell subsets, including CD20⁺ cells enriched for translational programs, CD19⁺_AP1, and CD27⁺ memory B cells, were elevated. Among these, only CD19⁺_AP1 had a higher frequency relative to post-treatment, indicating selective persistence of specific B-cell populations (Fig. 4C, D; Fig. S3D).

#### Innate lymphoid cells: translational enrichment in responders versus exhausted phenotypes in nonresponders

**Figure 5:**
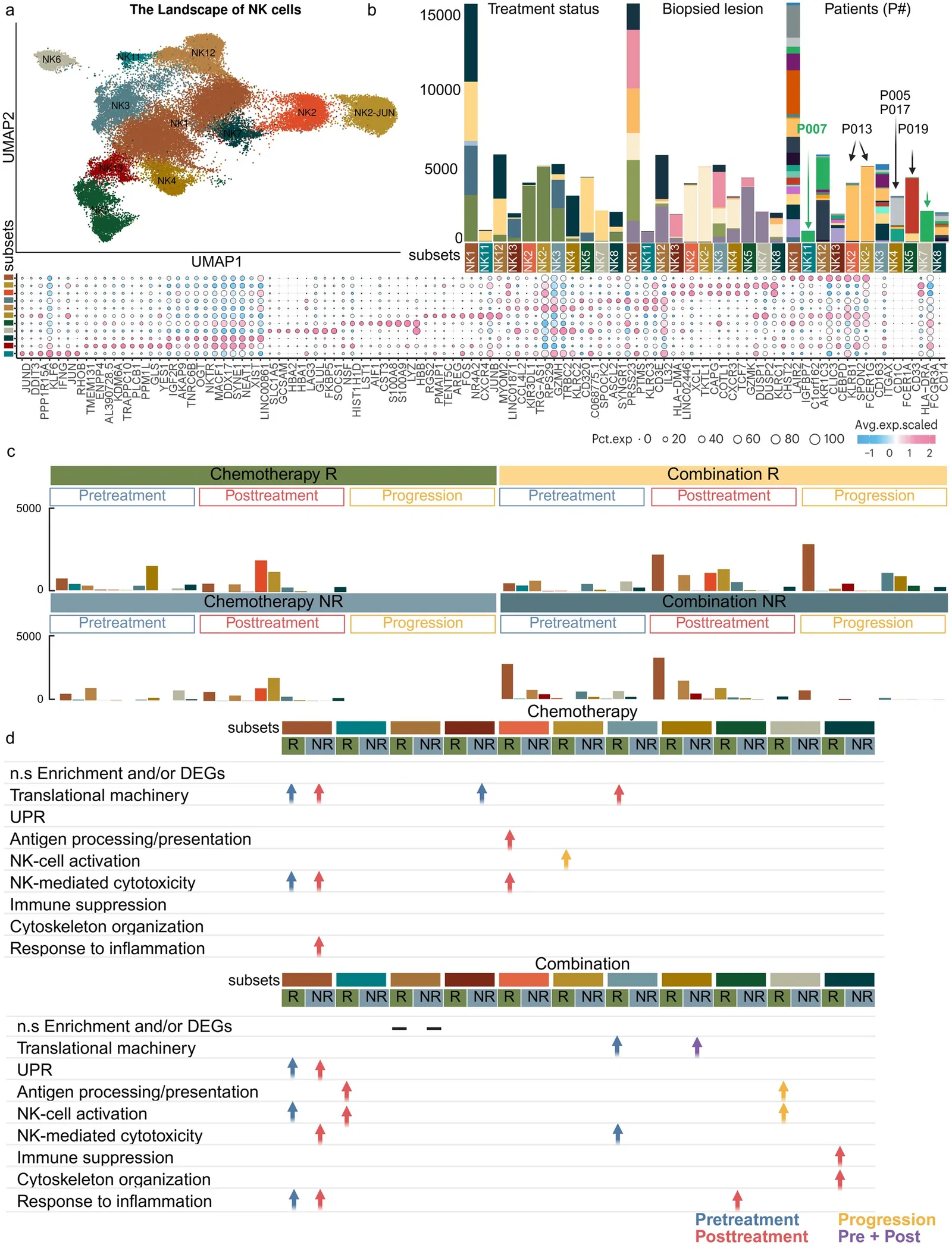
Landscape and dynamics of NK cell subsets in blood across treatment conditions. (A) UMAP of NK cell sub-clusters in blood, colored by functional states. Bubble plot shows representative marker gene expression across clusters. Additional marker genes are listed in Table S2. (B) Stacked bar plots showing NK sub-cluster distributions across treatment groups, response status, biopsied lesions, and individual patients, using color annotations consistent with Fig. 1B. (C) Bar plots comparing NK sub-cluster compositions before and after treatment. (D) Table summarizing enriched pathways or functional programs by sub-cluster and condition. Blue: enriched in pretreatment; red: enriched in post-treatment; yellow: enriched in progression; purple: enriched in both pre- and post-treatment. This figure highlights the heterogeneity of NK cells, their patient-specific patterns, and dynamic functional shifts in response to treatment. Enrichment analyses reveal key immune pathways underpinning NK-cell responses in the blood. avg.exp., average expression; n.s., not significant; NR, nonresponder; pct.exp., percentage of cells expressing the gene; R, responder; UPR, unfolded protein response.

NK cells are innate lymphocytes that mediate rapid, Ag-independent cytotoxic responses against tumor cells while producing cytokines that shape adaptive antitumor immunity. Of the 13 NK cell clusters identified (Table S2), 3 functional clusters (NK1, NK3, and NK8) that were representative across patients were selected for detailed analysis (Fig. 5A, B). In chemotherapy responders, NK1 cells had elevated translational activity and numbers of cytotoxic markers at baseline, suggesting an innate readiness that parallels CD8^+^ T-cell activation (Fig. 5C, D). This coordinated priming reflects classical NK cell function, part of the broader family of group 1 innate lymphoid cells, which are known to provide rapid effector responses and support adaptive immunity.^13^ In contrast, combination therapy responders had enhanced innate engagement after treatment, with expansion of NK1 cells with elevated translational and inflammatory signaling, indicative of heightened NK cell activation and functional differentiation (Fig. 5C, D).

In chemotherapy nonresponders, treatment induced an increase in NK1 cells enriched for translational machinery, inflammatory response, and cytotoxicity or exhaustion markers, whereas NK3 subsets were enriched for translational activity (Fig. 5C, D). Collectively, these patterns suggest chemotherapy in nonresponders promotes immune subset expansion, but this is skewed toward a dysregulated, pro-inflammatory, and exhausted phenotype lacking coordinated effector function.^27^ Similarly, in combination-therapy nonresponders, NK3 cells at baseline exhibited cytoplasmic translation enrichment and inhibitory markers (*KIR2DL3*, *KLRG1*) consistent with an exhausted phenotype (Fig. 5C, D). After treatment, NK1 cells showed increased ER stress, inflammatory response, and cytotoxic or exhaustion signatures, paralleling the chemotherapy nonresponder profile (Fig. 5C–E).

These findings highlight distinct patterns of immune dysregulation between treatment groups. In chemotherapy nonresponders, CD8^+^ T-cell diversity expanded but lacked coordinated functional engagement with an elevated exhaustion phenotype. In contrast, combination-treated nonresponders exhibited more defined CD8^+^ activation but were marked by CD4^+^ stress responses, suggesting dysfunctional immunity. This aligns with observations of a pan-cancer CD4⁺ T-cell stress–response state linked to immunotherapy resistance.^28^ Notably, translationally active CD20^+^ and CD27^+^ B-cell subsets remained elevated after treatment but lacked functional enrichment, suggesting limited contribution to effective immunity. Both treatments resulted in elevated exhaustion levels.

### Myeloid cells

Myeloid responses showed treatment- and response-specific temporal immune programs. Chemotherapy responders displayed pretreatment enrichment of metabolically active, Ag-presenting myeloid cells, whereas combination-treatment responders exhibited post-treatment expansion of pro-inflammatory monocytes. Nonresponders to both regimens had post-treatment metabolic activation without effective inflammatory programs, paralleling dysfunctional lymphoid immune programs.

#### Divergent timing of myeloid immune programs in responders to chemotherapy and combination therapy

**Figure 6:**
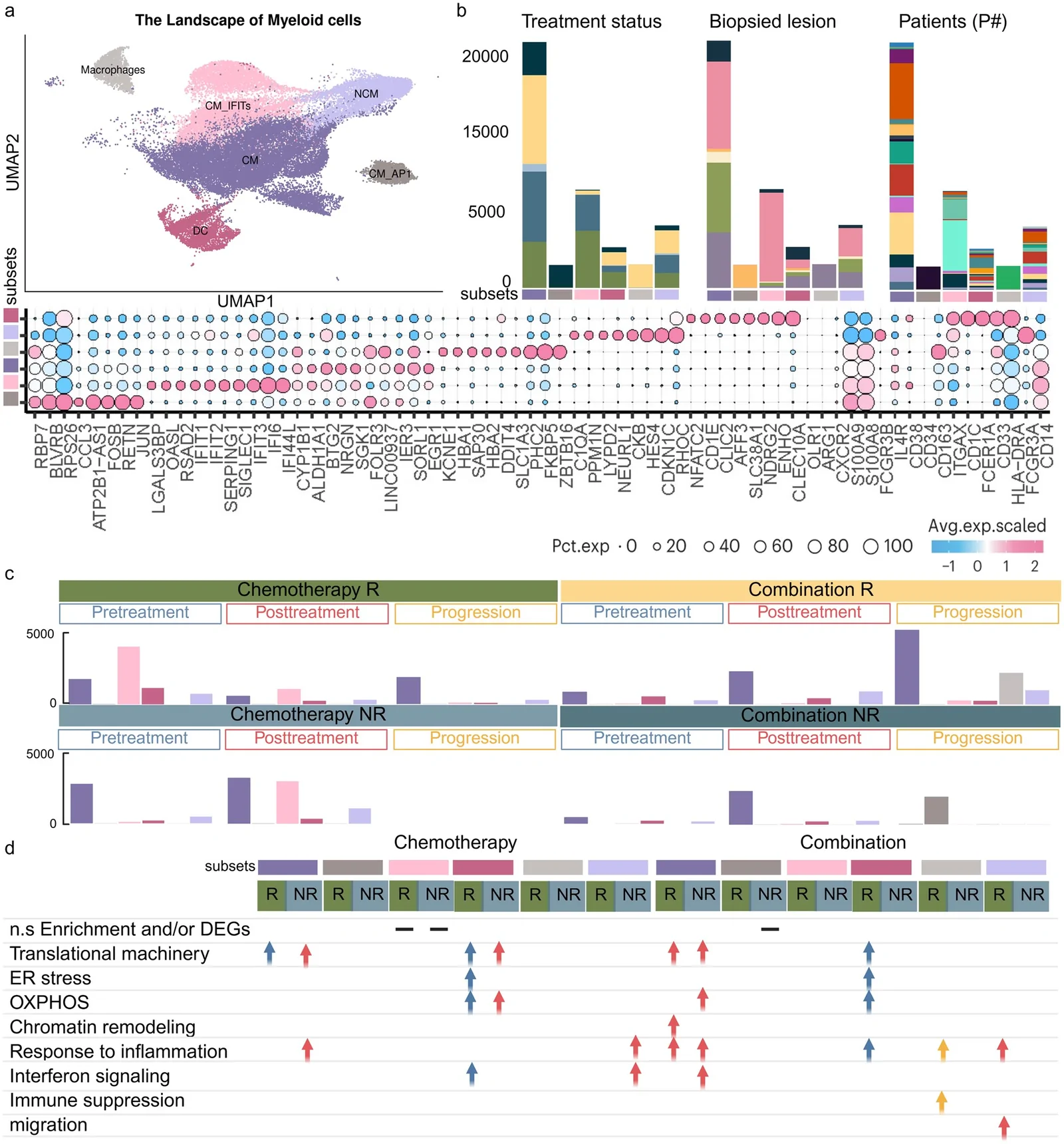
Landscape and dynamics of monocyte subsets in blood across treatment conditions. (A) UMAP of monocyte sub-clusters in blood, colored by distinct functional states. The bubble plot shows representative marker gene expression; additional markers are listed in Table S2. (B) Stacked bar plots showing monocyte sub-cluster distributions across treatment groups, response status, biopsied lesions, and individual patients (P#), using color annotations consistent with Fig. 1B. (C) Bar plots comparing monocyte sub-cluster compositions before and after treatment. (D) Table summarizing enriched pathways or functional programs by sub-cluster and condition. Blue: pretreatment; red: post-treatment; yellow: progression. This figure highlights the heterogeneity of circulating monocytes, their patient-specific distribution patterns, and dynamic shifts in composition and function following treatment. Enrichment analyses reveal pathways underlying monocyte responses in the peripheral blood. avg.exp., average expression; n.s., not significant; NR, nonresponder; R, responder; pct.exp., percentage of cells expressing the gene.

Myeloid populations coordinate Ag presentation, inflammatory signaling, and tissue remodeling, but can also acquire immunosuppressive and pro-tumorigenic phenotypes. Of the 9 myeloid clusters identified (Table S2), 3 functional clusters (classical monocyte [CM], nonclassical monocyte [NCM], and dendritic cells [DCs]) that were representative across patients were selected for detailed analysis (Fig. 6A, B). In the chemotherapy group, pretreatment samples from responders showed increased frequencies of CMs enriched for translational machinery, and DCs enriched for translational machinery, ER stress, OXPHOS, and IFN signaling. This transcriptional profile is consistent with a metabolically active, Ag-presenting phenotype (Fig. 6C, D; Fig. S5A, B). This suggests preexisting immune activation, particularly metabolically active and Ag-presenting myeloid cells, is necessary for an effective response to chemotherapy.^6^ Together with adaptive immune priming, this also suggests an interplay between the adaptive and innate immunity, priming the microenvironment for tumor clearance. However, no significant expansion of myeloid cells was observed following chemotherapy treatment, similar to the lack of expansion observed in lymphoid cell types. In the combination-therapy group, pretreatment samples from responders had increased DC infiltration, with the transcriptional landscape showing enrichment for translational machinery, stress, and inflammatory response (Fig. 6C, D; Fig. S5A, B). After treatment, responders exhibited an expansion of CMs enriched for chromatin remodeling, suggesting a memory-like phenotype with a pro-inflammatory transcriptional program (Fig. 6C, D; Fig. S5A, B). NCMs were enriched for a cytotoxic, IFN-responsive, and migratory phenotype, including *ITGA4*, *TLN1*, *MSN*, and *CORO1B* markers (Fig. 6C, D; Fig. S5A, B). These findings, in parallel with reduced adaptive immunity, are consistent with robust immune remodeling after anti–PD-L1 therapy.

#### Myeloid compartment in nonresponders exhibits inflammatory skewing and immune dysfunction

Pretreatment, nonresponder samples across both treatment groups lacked higher myeloid presence than post-treatment samples. After chemotherapy, nonresponders had an increased frequency of CMs enriched for cytoplasmic translation, inflammatory signaling, and wound-healing pathways (*PF4*, *PF4V1*, *SPARC*, *TGFB1*), indicative of a pro-tumorigenic and potentially immunosuppressive state. NCMs had upregulation of inflammatory and type I IFN response programs, and DCs exhibited elevated cytoplasmic translation and OXPHOS, reflecting heightened metabolic activity (Fig. 6C, D; Fig. S5A, B). After treatment, nonresponders displayed a myeloid compartment dominated by metabolically active, pro-tumorigenic programs, in line with evidence that tumor metabolic reprogramming can induce suppressive myeloid phenotypes.^29^^,^^30^ Concurrently, diversity in the lymphoid compartment was increased, with features suggestive of T-cell dysfunction or exhaustion. This pattern is consistent with delayed and ineffective immune activation, potentially reflecting tumor-driven immune dysregulation. A similar escape mechanism has been described in the context of tumor-mediated disruption of Ag presentation of T-cell exhaustion as a mode of cancer immune evasion.^31^ In the combination group, post-treatment CMs upregulated cytoplasmic translation, OXPHOS, type I IFN signaling, and immunoregulatory genes (*SOCS3*, *BCL3*), suggesting activation skewed toward dysregulation rather than effective antitumor immunity^32^ (Fig. 6C, D; Fig. S5A, B). NCMs showed elevated cytoplasmic translation but lacked hallmark inflammatory or cytotoxic gene expression (Fig. 6C, D; Fig. S5A, B), indicating a metabolically primed state without clear evidence of functional activation. Such metabolic reprogramming without effector function is a recognized pattern in tumor-associated myeloid cells, where translation-driven activation contributes to suppression rather than immune effector activity.^33^ In parallel, the lymphoid compartment exhibited an increase in terminally exhausted T cells, further reinforcing a state of ineffective immune activation in nonresponders.

### Tissue CD4^+^/CD8^+^ T-cell dynamics diverge in nonresponders

**Figure 7:**
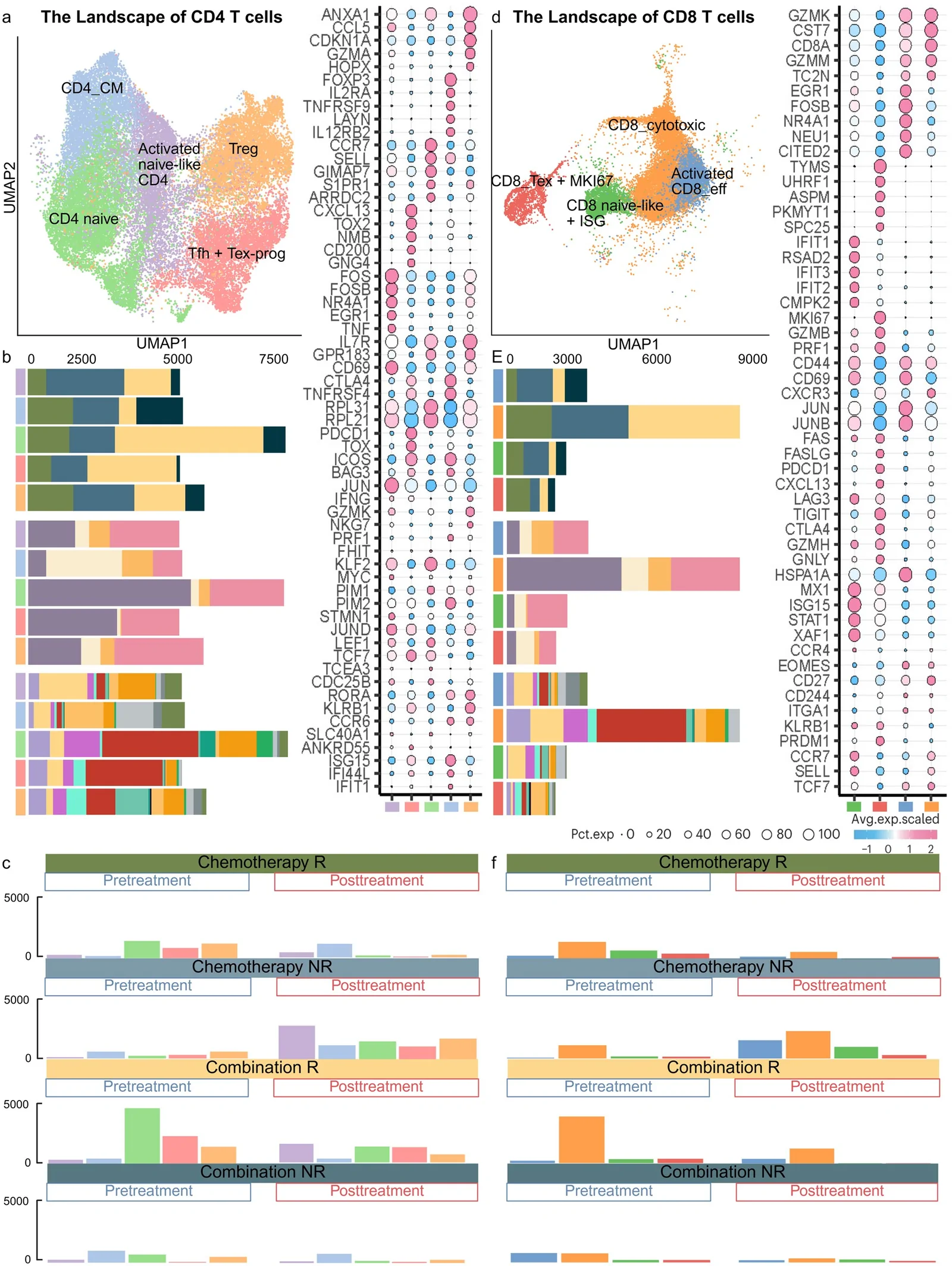
Comprehensive landscape of CD4^+^ and CD8^+^ T-cell subsets in tissue samples and their distribution across conditions. (A–D) UMAP representation of CD4^+^ and CD8^+^ T-cell sub-clusters in tissue, respectively, colored by distinct functional states. The bubble plot shows representative marker gene expression; additional markers are listed in Table S2. (B–E) Stacked bar plots showing CD4^+^ and CD8^+^ sub-cluster distributions, respectively, across treatment groups, response status, biopsied lesions, and individual patients (P#), using color annotations consistent with Fig. 1B. (C)–(F) Bar plots comparing T-cell sub-cluster compositions before and after treatment. avg.exp., average expression; n.s., not significant; NR, nonresponder; pct.exp., percentage of cells expressing the gene; R, responder.

Of the 7 CD4^+^ and 14 CD8^+^ T-cell clusters identified (Table S2), 5 CD4^+^ and 4 CD8^+^ functional clusters that were representative across patients were selected for detailed analysis (Fig. 7A, B, D, E). Pretreatment, both chemotherapy and combination therapy responders showed enrichment of adaptive immune subsets, including CD4_naive, Tfh + Tex-prog, Treg, CD8_cytotoxic, CD8_Tex + MKI67, and CD8_naive-like + ISG populations, indicating a preexisting, Ag-experienced T-cell compartment poised for activation (Fig. 7C, F). These observations, that identified naïve T cells, Tfh-CXCR5, and Tex-MKI67 subsets were enriched pretreatment in PTX responders but appeared post-treatment in nab-PTX responders, align with those of Zhang et al.^7^^,^^8^ After treatment, both groups showed increased activated naïve-like CD4^+^ T cells, whereas combination therapy resulted in increased activated CD8-eff and chemotherapy resulted in increased CD4-CM levels (Fig. 7C, F). Zhang et al.^7^^,^^8^ showed that activated naïve-like CD4 was associated with combination post-treatment in PTX, but with pretreatment in nab-PTX. In nonresponders, pretreatment profiles diverged by treatment. Combination-therapy nonresponders showed enrichment of CD4_naïve, CD4_CM, Treg, activated CD8_eff, and CD8_cytotoxic subsets (Fig. 7C, F). Chemotherapy nonresponders instead exhibited broad T-cell expansion after treatment (Fig. 7C, F), consistent with the observation by Zhang et al.^7^^,^^8^ of postchemotherapy increases in Treg cells.

### Coordinated systemic and tumor T-cell programs distinguish chemotherapy and combination-therapy responders

**Figure 8:**
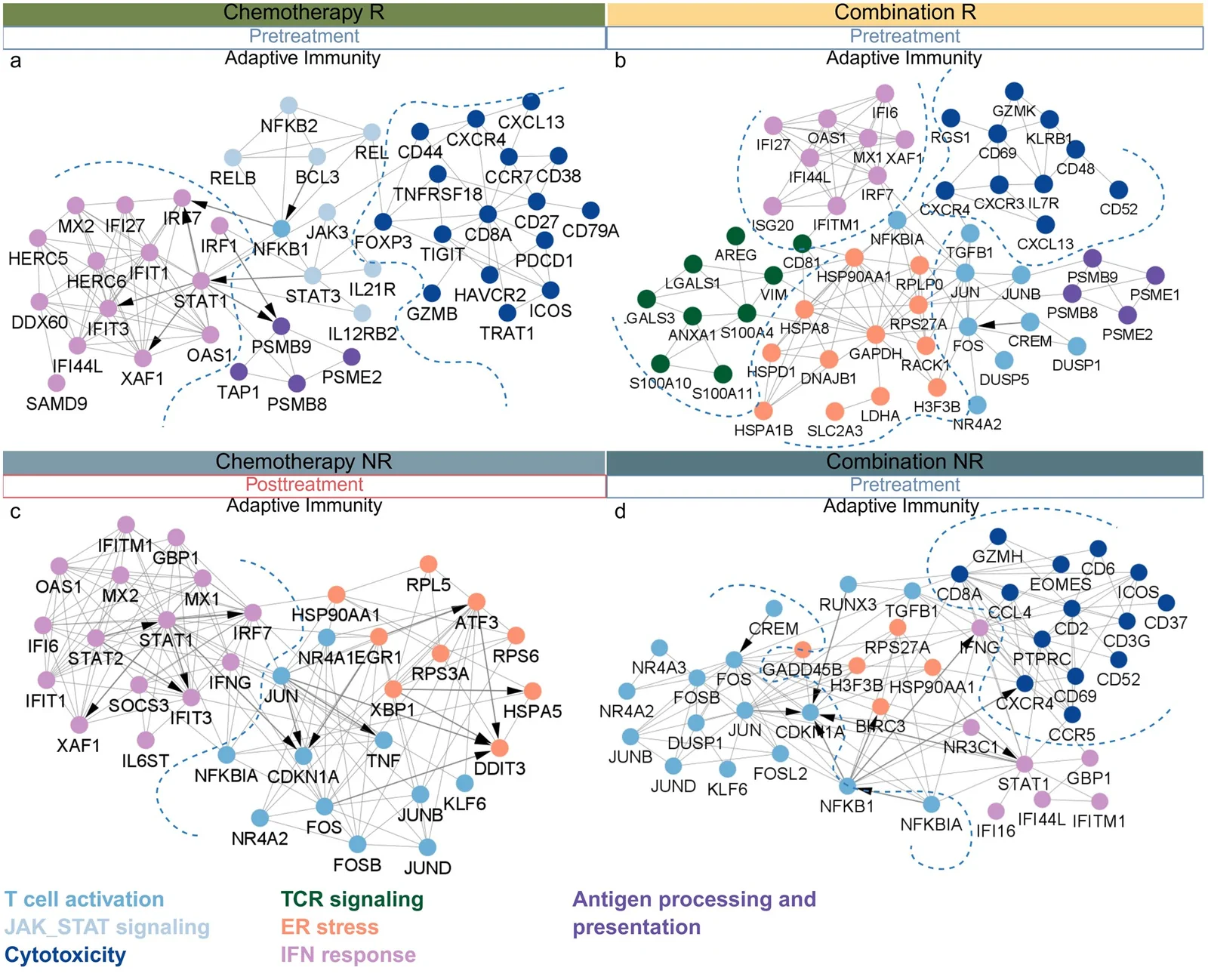
PPI networks of treatment- and response-specific mechanisms in tissue. PPI networks were constructed from DEGs identified in each treatment and response group when comparing post- and pretreatment samples. (A) Chemotherapy responders (R) (pretreatment): modules enriched in T-cell activation, Ag processing, IFN signaling, and cytotoxicity. (B) Combination-therapy responders (pretreatment): modules enriched in IFN signaling, T-cell activation, endoplasmic reticulum (ER) stress, and TCR signaling. (C) Chemotherapy nonresponders (NR) (post-treatment): modules enriched in T-cell activation, ER stress, and IFN response. (D) Combination-therapy nonresponders (pretreatment): modules enriched in T-cell activation, IFN signaling, ER stress, and cytotoxicity. Node color denotes functional modules and arrows indicate transcription-target relationships. Together, these networks reveal condition-specific immune regulatory circuits and highlight potential therapeutic targets.

**Figure 9:**
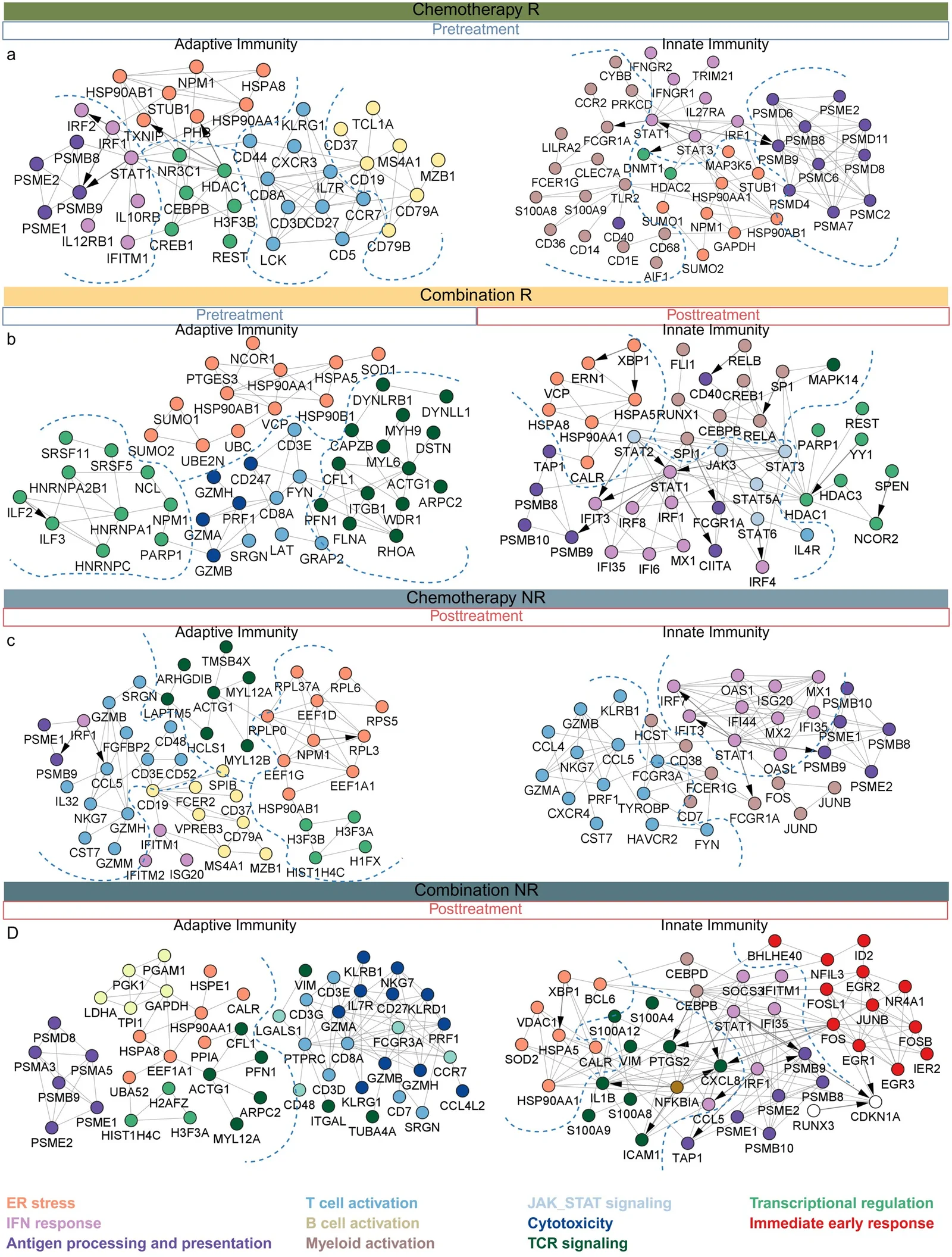
PPI networks of treatment- and response-specific mechanisms in blood. PPI networks were constructed from DEGs identified in each treatment and response group when comparing post- and pretreatment samples. (A) Chemotherapy responders (R) (pretreatment): adaptive immunity (left panel) modules enriched in T-cell activation, Ag processing, IFN signaling, and transcriptional regulation. (B) Innate immunity (right panel) modules enriched in myeloid activation, IFN signaling, endoplasmic reticulum (ER) stress and Ag processing. Combination responders (pretreatment): adaptive (left panel) modules enriched in T-cell activation, cytotoxicity, ER stress, and transcriptional regulation. Combination-therapy responders (post-treatment): innate immunity (right panel) modules enriched in myeloid activation, IFN signaling, ER stress, and transcriptional regulation. (C) Chemotherapy nonresponders (post-treatment): adaptive immunity (right panel) modules enriched in T- and B-cell activation, TCR signaling, ER stress, and IFN response. Innate immunity (right panel), myeloid activation, JAK-STAT signaling, Ag processing, and IFN signaling. (D) Combination-therapy nonresponders (post-treatment): adaptive immunity (left panel) modules enriched in T-cell activation, IFN signaling, ER stress, and cytotoxicity. Innate immunity (right panel) modules enriched in motility, Ag presentation, IFN signaling, ER stress, and immediate early response. Node color denotes functional modules and arrows indicate transcription-target relationships. Together, these networks reveal condition-specific immune regulatory circuits and highlight potential therapeutic targets.

In chemotherapy responders, tissue PPI network analysis revealed enrichment of IFN-driven signaling, Ag processing, and strong exhaustion markers (Fig. 8A). Blood profiling showed upregulation of T-cell priming or activation states and early activation of CD8^+^ T cells (Fig. 9A). This concordance suggests a sequential immune mobilization model in which activated T cells detected in the blood are associated with intratumoral cytotoxic and exhaustion programs. In combination therapy responders, tissue networks highlighted ribosomal proteins, metabolic enzymes, and TCR activation markers, consistent with sustained Ag-driven effector function supported by high biosynthetic and metabolic capacity (Fig. 8B). Pretreatment blood profiles mirrored this state, with more fully differentiated and engaged adaptive immunity with active TCR signaling and cytotoxicity responses (Fig. 9B), indicating parallel activation of systemic and intratumoral T-cell compartments at treatment onset. Notably, post-treatment blood, in combination therapy, shifted toward expansion of innate immune subsets with JAK–STAT, TNF, and IFN pathway activation. These observations are consistent with systemic, innate immune re-engagement that may follow effective adaptive tumor clearance, even if not captured in matched tissue samples, due to lack of representative innate immunity across patients (Fig. 9B).

In chemotherapy nonresponders, post-treatment showed tissue enrichment for Ag-driven activation and effector differentiation, as well as stress-response and immunoregulatory programs (*TNF*, *TNFSF10*, *IL6ST*, *NFKBIA*, *SOCS3*, and *ZC3HAV1*) (Fig. 8C). Blood analysis revealed parallel induction of effector, cytotoxicity, and exhaustion markers, reinforcing the interpretation that transcriptionally primed T cells remain functionally constrained (Fig. 9C). In tissue, pretreatment combination-therapy nonresponders had PPI networks combining Ag-driven activation, cytotoxic function, and metabolic adaptation, but these were accompanied by immunoregulatory and stress-response signatures (*TNFAIP3*, *BIRC3*, *TGFB1*, *NR3C1*, *NFKBIA*) and chemokine/cytokine modulators (*CCR5*, *CXCR4*, *CCL4*) (Fig. 8D), suggesting functional suppression within an immunosuppressive microenvironment.^34^ This suppression was mirrored in blood, where reduced effector signatures were observed (Fig. 3). Across both therapies, nonresponders demonstrated post-treatment expansion of innate immune subsets in blood, characterized by oxidative stress, metabolic dysfunction, and immunosuppressive signaling. PPI analysis indicated upregulation of antioxidant and inflammatory regulators (*SOD2*, *PRDX*, *HMOX1*) alongside *NFKBIA* and *PTGS2*, with stronger induction in combination-therapy nonresponders, suggesting unresolved inflammation and dysfunctional innate immune re-engagement (Fig. 9C, D).

### Patient-specific immune dynamics

Although group-level analyses revealed broad immune differences between responders and nonresponders, patient-specific analyses uncovered nuanced immune adaptations shaped by metastatic site and therapeutic response. In chemotherapy responders, patients P020 (breast), P022 (chest wall metastasis), and P013 (liver metastasis) had distinct myeloid and NK cell dynamics (Fig. 5B, Fig. 6B). In blood, a pretreatment increase in CM_IFIT cells was consistently observed across these patients, reflecting a shared inflammatory signature (Fig. 6C). Interestingly, in patient P013, treatment was followed by an increase of NK2 and, at progression, increased by NK2_JUN, both involved in processing and presenting Ags, suggesting a dynamic NK cell response (Fig. 5C–E). Tissue profiling of patient P013 revealed a sequential immune trajectory. Pretreatment pro-inflammatory monocytes transitioned into pro-inflammatory macrophages after treatment, followed by increased NK1, NK2, TAM + ISG, macrophages, and macrophage + LYVE1 (Fig. S6A, C) (Fig. S7A, C). These populations had elevated expression of inflammatory and cytokine response genes, emphasizing the role of innate immunity in mediating chemotherapy response in metastatic disease^35^ (Fig. S6D, and Fig. S7D). Notably, pretreatment adaptive immune engagement, characterized by primed CD4^+^ T cells and diverse CD8^+^ effector subsets, preceded the expansion of innate immune populations, suggesting a coordinated interplay in which early adaptive activation primes subsequent innate immune responses during treatment and disease progression.

In chemotherapy nonresponders, blood samples from patients P018, P023, P024, and P025 (all with breast cancer) showed a post-treatment increase in CM_IFIT, aligning with the inflammatory profile observed at the group level (Fig. 6C). In combination-therapy responders, patients P019 and P007 (lymph node metastases) exhibited distinct immune dynamics. In blood, both patients had post-treatment increases in NK5 and NK11, subsets associated with NK activation and Ag processing, consistent with group-level observations and highlighting innate immune activation after combination therapy (Fig. 6C, D). These observations align with group-level findings, reinforcing the role of innate lymphoid immune activation after combination therapy. At progression, NK6, NK12, and macrophage populations were elevated, indicating ongoing immune adaptation (Fig. 6C). In contrast, combination-therapy nonresponders, including P005 and P017 (both with liver metastases), had distinct pretreatment immune features. In PBMC analysis, NK4 emerged in a patient-specific manner, present before treatment in patient P005 and after treatment in patient P017, suggesting differential NK-cell adaptation (Fig. 5B, C). Tissue profiling of patient P017 revealed pretreatment enrichment of NK5, characterized by cytoplasmic translation and cytoskeleton organization (Fig. S6B and C), alongside CMs characterized with early immune activation, which were replaced after treatment by macrophage + HS3ST2–expressing immunosuppressive markers (Fig. S7B and C). Patient P005 had NK3 cells enriched for cytoplasmic translation pretreatment (Fig. S6B and C). Additionally, patient P016 (with chest wall metastasis) had increased NK12 before treatment, with NK7–expressing cytoskeleton organization and checkpoint markers (Fig. S6B and C), and emergence of CM_AP1 emerging at progression in PBMCs (Fig. 6B). Tissue samples revealed early macrophage + CCL3 enrichment, suggesting a preexisting myeloid-driven immune phenotype (Fig. S7B and C). Collectively, these findings suggest innate immune subsets with immunosuppressive features are associated with poor response to combination therapy.

Together, these findings highlight the heterogeneity of immune responses across patients and therapeutic regimens. Although adaptive immunity appears to play a critical role in initiating response, the dynamic engagement or suppression of innate immune subsets during treatment and progression may be key to sustaining or modulating therapeutic efficacy. These results underscore the importance of incorporating patient-specific immune profiling to refine stratification strategies and inform the development of individualized therapeutic approaches.

### Extension to I-SPY2 clinical trial data

To contextualize these findings against publicly available datasets, we leveraged scores derived from the I-SPY2 clinical trial.^11^ Correlation analysis demonstrated that NK1, Treg, and activated or exhausted CD8 programs positively aligned with tumor chemokine, IFN, and T- and B-cell modules, whereas proliferating ISG-CD8 programs had a consistent negative association, indicating concordance between systemic immune states and established tumor immune phenotypes (Fig. S8A). To maintain treatment consistency, analyses were restricted to the pretreatment PTX arm. Univariate logistic regression showed directional but underpowered associations with pathological complete response. In contrast, multivariable LASSO modeling identified a coordinated peripheral immune signature associated with response, negative contributions from activated naïve-like CD4 and DC programs, and positive contributions from NK, naïve CD8, and Treg states, suggesting composite systemic immune readiness may distinguish responders (Fig. S8B). Permutation testing indicated that the multivariate model performed better than random expectation but did not reach statistical significance (*P* = 0.128), which was consistent with the limited cohort size and correlated immune features.

## Discussion

This study provides a systematic characterization of immune-cell populations and their associated mechanisms, revealing shared and distinct immunological features underlying treatment response and resistance in patients with advanced TNBC who were receiving chemotherapy or chemoimmunotherapy. Peripheral immune dynamics differed by treatment and response, revealing therapy-specific immunological states with predictive and mechanistic interpretability. Chemotherapy responders had higher pretreatment immune-cell counts than did combination-therapy responders, followed by post-treatment depletion consistent with known cytotoxic effects.^36^^,^^37^ In nonresponders, those receiving combination therapy had higher pretreatment counts than the chemotherapy-only group, but immune levels remained elevated after treatment across groups. These findings are concordant with recent I-SPY2 peripheral blood data indicating that therapeutic response is linked to systemic immune activation and cytotoxic remodeling in chemoimmunotherapy.^12^

Across both treatment groups, our results highlight the importance of the dynamic coordination between adaptive and innate immune programs in modulating treatment outcomes. Enrichment of pathways, including cytoplasmic translation, stress response, inflammatory signaling, cytoskeletal remodeling and migration, and metabolic programs including OXPHOS were observed in both responders and nonresponders, underscoring their association with treatment outcomes. Post-transcriptional mechanisms, including the translation of mRNA, are critical contributors to rapid adaptive changes in the cellular proteome during immune responses.^38^ For instance, cytosolic protein translation is essential to increase glucose metabolism and degranulation capacity upon TCR activation, regulating the effector functions of cytotoxic T lymphocytes and influencing the formation of memory cells.^37^^,^^39^ Additionally, reprogramming cellular protein synthesis in innate immunity plays a pivotal role in inflammatory responses to pathogens and tumorigenesis by enhancing DNA-dependent cyclic GMP-AMP synthase (cGAS) activation, which drives the expression of ISGs.^40^

Chronic Ag exposure can induce PD-L1 expression in tumor cells (likely via IFN-γ secreted from T cells)^41^ and sustain PD-1 signaling in T cells, leading to an epigenetically imprinted exhaustion program.^42–44^ The presence of more differentiated T cells in combination-therapy responders reflects this exhaustion phenotype and suggests higher tumor PD-L1 levels, potentially enhancing sensitivity to anti–PD-L1 therapy. This was supported by our observation that in responders, tumors in combination therapy were PD-L1^+^, whereas in nonresponders they were PD-L1^−^. In contrast, chemotherapy responders had less differentiated and more plastic T cells before treatment, consistent with lower PD-L1 expression and increased chemotherapy responsiveness. Combination therapy induced increased innate immune engagement, potentially in response to increased antigenic debris from tumor cell death. Such innate activation is critical for sustaining T-cell memory and effector function, linking Ag presentation and cytokine signaling to adaptive immune maintenance.^36^ Innate immune reactivation after combination therapy may serve as a surrogate marker of response. Overall, these results provide a system-level context for previous studies showing that pretreatment immune-cell diversity plays a crucial role in treatment outcomes.^37^^,^^39^^,^^45^

In contrast, nonresponders exhibited a myeloid compartment skewed toward an immunoregulatory, metabolically reprogrammed state, paralleled by increased dysfunctional and exhausted lymphoid populations across both treatments. Although canonical myeloid-derived suppressor cell (MDSC) markers such as ARG1 were not prominently expressed, IL-1B was present among DEGs and, coupled with upregulation of stress-adaptive and metabolic genes, this profile is consistent with an early-stage or atypical MDSC-like phenotype.^46^ This immunoregulatory shift may be associated with CD4⁺ T-cell polarization toward suppressive (Treg) or inflammatory (Th17) fates, as reported for metabolically reprogrammed human myeloid subsets.^47^ Such metabolically primed myeloid cells have been shown to suppress T- and NK-cell activity or promote chronic inflammation, consistent with studies implicating monocyte and stromal metabolic reprogramming via cGAS-STING as a driver of therapy resistance.^48^ These findings highlight the plasticity of tumor-associated myeloid cells and the emerging importance of immunometabolism in shaping protumoral functions.^17^^,^^46^

Dysregulated NK-cell responses emerged as a converging axis of resistance, with increased expression of DUSP1, CD81, and cytotoxic stress markers. Prior studies in TNBC have linked persistent NK activation with poor prognosis, with inflammatory NK signatures being increasingly recognized as pro-metastatic in solid tumors.^47^^,^^49–51^ Together, these data support a model in which adaptive-innate immune interplay in the peripheral blood is a marker and correlate of therapeutic outcome. Responders were characterized by coordinated activation of lymphoid and metabolically competent myeloid populations, whereas nonresponders had myeloid immunoregulation and lymphoid dysfunction or exhaustion. These results further highlight the potential of peripheral immune profiling to guide response prediction, resistance monitoring, and immunomodulatory strategies in TNBC.

In conclusion, our single-cell profiling of paired blood and tumor samples reveals that systemic and intratumoral immune remodeling distinguishes therapeutic responders from nonresponders in TNBC. Responders exhibit coordinated activation of adaptive and innate immune programs, whereas nonresponders display metabolic reprogramming and immune dysfunction across compartments. The implications of our study include potential development of blood-based biomarkers associated with treatment response, informing patient-specific therapeutic strategies and the rational design of more precise combination therapies to increase response to treatment in patients with TNBC.

## Supplementary Material

vlag049_Supplementary_Data

## Data Availability

All datasets analyzed in this study are publicly available in the Gene Expression Omnibus under accession number GSE169246. Processed data and analysis scripts are available from the corresponding author upon request.
