# Supplementary material for: Differential peripheral immune dynamics underlie therapeutic response to chemotherapy and chemoimmunotherapy in triple-negative breast cancer
Source: Immunohorizons. 2026 Sep 22;10(9):vlag049. doi: 10.1093/immhor/vlag049 (PMC13597118; doi:10.1093/immhor/vlag049)
Supplement: Supplementary file 1 [file vlag049_supplementary_data.zip › SupplementaryMaterial.docx]

**Supplementary Material:**

**Supplementary Figure S1. Comparative immune landscape of tissue samples under different treatment conditions.**

**(A)** UMAP plots depict major immune cell types in tissue, highlighting T cells (blue), B cells (yellow), monocytes (pink), and NK cells (brown). **(B)** Compositions of major immune cell types in treatment and response groups, biopsied lesions, and individual patients. **(C)** Bar plots quantifying immune cell frequencies, illustrating treatment-associated shifts in immune cell distribution. **(E)** Regression model sensitivity analysis showing the impact of removing each patient on model fit (REML) (top left, pretreatment; bottom left, posttreatment) and stability of fixed-effect estimates (β) across leave-one-out iterations (top right, pretreatment; bottom right, posttreatment).

**Supplementary Figure S2. CD4+ T-cell transcriptional dynamics in blood across treatment response groups.**

**(A)** Heatmap showing median changes in CD4⁺ T-cell functional module scores between posttreatment and pretreatment samples across CD4⁺ T-cell subsets and treatment-response groups. Red indicates increased scores post-treatment relative to pre-treatment, and blue indicates decreased scores. Asterisks denote statistical significance. **(B)** Dot plot summarizing log₂ fold-change (post/pre) in abundance of CD4⁺ T-cell subsets across treatment-response groups; dot size represents absolute fold change. **(C)** Pathway enrichment analysis of representative CD4⁺ T-cell subsets in blood. Red indicates significantly upregulated pathways, blue indicates downregulated pathways, and grey indicates non-significant enrichment. Significance is shown as signed –log10(adjusted p value).

**Supplementary Figure S3. CD8+ T-cell and B-cell transcriptional dynamics in blood across treatment response groups.**

**(A)** Heatmap showing median changes in CD8⁺ T-cell functional module scores between posttreatment and pretreatment samples across CD8⁺ T-cell subsets and treatment-response groups. Red indicates increased scores post-treatment relative to pre-treatment, and blue indicates decreased scores. Asterisks denote statistical significance. **(B)** Dot plot summarizing log₂ fold-change (post/pre) in abundance of CD8⁺ T-cell subsets across treatment-response groups; dot size represents absolute fold change. **(C)** Pathway enrichment analysis of representative CD8⁺ T-cell subsets in blood. Red indicates significantly upregulated pathways, blue indicates downregulated pathways, and grey indicates non-significant enrichment. Significance is shown as signed –log10(adjusted p value). **(D)** Dot plot summarizing log₂ fold-change (post/pre) in abundance of B-cell subsets across treatment-response groups; dot size represents absolute fold change.

**Supplementary Figure S4. Natural killer cell transcriptional dynamics in blood across treatment response groups.**

**(A)** Pathway enrichment analysis of representative NK cell subsets in blood. Red indicates significantly upregulated pathways, blue indicates downregulated pathways, and grey indicates non-significant enrichment. Significance is shown as signed –log10(adjusted p value). **(B)** Dot plot summarizing log₂ fold-change (post/pre) in abundance of NK cell subsets across treatment-response groups; dot size represents absolute fold change.

**Supplementary Figure S5. Myeloid cell transcriptional dynamics in blood across treatment response groups.**

**(A)** Pathway enrichment analysis of representative myeloid cell subsets in blood. Red indicates significantly upregulated pathways, blue indicates downregulated pathways, and grey indicates non-significant enrichment. Significance is shown as signed –log10(adjusted p value). **(B)** Dot plot summarizing log₂ fold-change (post/pre) in abundance of myeloid subsets across treatment-response groups; dot size represents absolute fold change.

**Supplementary Figure S6.** **Comprehensive landscape of Natural Killer cell subsets in tissue samples and their distribution across conditions.**
**(A)** UMAP representation of NK cell sub-clusters in tissue, colored by distinct functional states. **(B)** UMAP plots showing patient-specific distributions of NK cell sub-clusters across multiple individuals. **(C)** Stacked bar plots depicting the relative abundance of NK cell sub-clusters in tissue across different conditions. **(D)** Bar plots comparing the composition of NK cell sub-clusters in tissue before and after treatment across multiple samples, colored by distinct functional states. (**F)** Table representation of enriched pathways or functional signatures associated with NK cell sub-clusters, stratified by condition. Blue: enriched in pretreatment. Red: enriched in posttreatment.

**Supplement Figure S7.** **Comprehensive landscape of Myeloid cell subsets in tissue samples and their distribution across conditions.**
**(A)** UMAP representation of myeloid cell sub-clusters in tissue, colored by distinct functional states. (**B)** UMAP plots showing patient-specific distributions of myeloid cell sub-clusters across multiple individuals. **(C)** Stacked bar plots depicting the relative abundance of myeloid cell sub-clusters in tissue across different conditions. **(D)** Bar plots comparing the composition of myeloid cell sub-clusters in tissue before and after treatment across multiple samples, colored by distinct functional states. **(F)** Table representation of enriched pathways or functional signatures associated with myeloid cell sub-clusters, stratified by condition. Blue: enriched in pretreatment. Red: enriched in posttreatment.

**Supplement Figure S8.** **Peripheral immune signatures align with tumor immune programs and jointly associate with pCR.**

**(A)** Correlation heatmap between peripheral immune cell subset scores derived from PBMC profiling in this study and tumor immune module scores from the I-SPY2 cohort. Tumor scores represent previously defined chemokine, interferon/STAT1, and T/B-cell immune programs, while rows denote blood-derived immune subsets identified from our PBMC analysis. Positive correlations indicate concordant systemic tumor immune activation states, whereas negative correlations highlight opposing proliferative/ISG-biased CD8 programs. **(B)** Multivariate LASSO logistic regression was applied to baseline PBMC-derived immune subset scores and I-SPY2 pCR outcomes to identify a coordinated peripheral immune signature associated with response. Bars represent non-zero coefficients at λ_min from cross-validated glmnet models. Positive coefficients (orange) indicate subsets positively associated with pCR, whereas negative coefficients (blue) indicate inverse associations. Coefficients are shown on the log-odds scale; the dashed line denotes no association.
